# Virtual suturing skills learning: role of verbal versus written feedback in surgery rotation clerkship

**DOI:** 10.1080/10872981.2021.2023937

**Published:** 2022-01-07

**Authors:** Daniel Ardian Soeselo, Alex Kusanto, Nurliati Sari Handini, Jonny Setiawan, Irene Stephanie, Michael Lie, Annatasia Ayu Gitasaraswati

**Affiliations:** Department of Surgery at School of Medicine and Health Sciences, Atma Jaya Catholic University of Indonesia, Jakarta, Indonesia

**Keywords:** Virtual-learning, surgery, suturing, education, feedback

## Abstract

**OBJECTIVE:**

Onsite clinical skills learning is interrupted during the SARS-CoV-2 pandemic. This study aimed to compare the effectiveness of verbal versus written feedback in virtual suturing skills learning.

**DESIGN:**

Participants were randomly divided into two groups, one with verbal feedback and written feedback on the other. Each was instructed to perform a simple interrupted suture which was assessed both before (pre-test) and after (post-test) feedback was given. Both groups were given the same duration of time to learn from the feedback they received.

**SETTING:**

Students undergoing clinical rotation in Department of Surgery at School of Medicine and Health Sciences, Atma Jaya Catholic University of Indonesia.

**PARTICIPANTS:**

The eligibility of the participants are students who enrolled on virtual learning in the surgery rotation and have consented to the research and those who own basic surgical equipment at home. A total of 199 participants were enrolled, with 17 (8.55%) participants dropping out.

**RESULTS:**

Out of 182 participants, 66 (36.3%) were males and 116 (63.7%) were females. The group receiving verbal intervention showed a significant difference both in suturing skills checklist (Pre-test [M = 4.11, SD = 0.61] and post-test [M = 4.73, SD = 0.37]; t(85) = −10.63, p = 0.000) and Global Rating Scale (GRS) assessment (pre-test [M = 3.83, SD = 1.06] and post-test [M = 4.56, SD = 0.64]; t(86) = −8.10, p = 0.000). In the same way, the group receiving written intervention also showed a significant mean pre-test and pos-test difference in both assessment tools (Checklist assessment [Z = −7.93, p = 0.000]; GRS assessment pre-test [M = 3.42, SD = 0.73] and post-test [M = 4.20, SD = 0.83]; t(105) = −9.62, p = 0.000). Both verbal and written feedback had a large effect (effect size >0.8).

**CONCLUSIONS:**

Both verbal and written feedback provide a significant result in improving suturing skills in a virtual learning setting which may provide a solution to online clinical skills training.

## Introduction

Clinical skills are one of the primary competencies needed to be fulfilled by medical graduates, and medical faculties are recommended to facilitate the learning process [1]. To achieve the expected level of clinical skills, Simulation-Based Medical Education (SBME) was considered the best method, and many literature reviews had elaborate ways to deliver them effectively [2,3]. One of the essential components in SBME is effective and comprehensive feedback, which will affect students learning positively. Well-delivered feedback will increase the effectiveness of the learning process by highlighting student’s learning development while at the same time also providing information on weaknesses where improvements can be done [4]. However, since the first SARS-CoV-2 case was announced in Indonesia in March 2020, all universities and medical education centers were forced to adapt and modify their learning method. Long-distance learning using a video conferencing platform in a lecture setting was set by our university to bridge the learning process during this pandemic. The application of the video conferencing method without any face-to-face meeting will surely have an impact on learning, especially clinical skills learning. Not only clinical students lost their chances to ‘learning by doing, but academic staff also unable to observe their students and provide feedback on daily basis.

Early in this pandemic, we have tried to give suturing skills learning by online demonstration, but the lack of real-time feedback limits the efficacy of the learning method. While electronically generated feedback is possible to be produced and delivered to students during this pandemic, the efficacy and its process have not received sufficient review especially in the setting of clinical training in Indonesia.

In this study, we expect to investigate the efficacy of electronic feedback in the setting of virtual suturing skill training, and compare the effectivity of verbal versus written feedback in improving the outcome of training by comparing the effect size of the two feedbacks method. We also intend to share our experience in providing electronic feedback in suturing skills training in the era of SARS-CoV-2 pandemic. We hypothesize that feedbacks will increase efficacy in virtual skills learning.

## Material and methods

### Sample size and criteria

This is an interventional study aiming to compare the efficacy of verbal and written feedback in virtual suturing skills learning. During the SARS-CoV-2 pandemic, the clinical rotation program in our university enacted virtual learning which occurred for two weeks in each department. The study is conducted in The Department of Surgery at The School of Medicine and Health Sciences of Atma Jaya Catholic University of Indonesia from March 2020 to August 2020 with the study size calculated by a formula recommended by previous research in medical education research with a final number of 60 participants [5]. Sample will be obtained through a consecutive sampling method on the clinical students enrolling in the Surgery clinical rotation at The School of Medicine and Health Science of Atma Jaya Catholic University of Indonesia.

The eligibilities of the participant are students who enrolled on virtual learning in the surgery rotation and have consented to the research, while participants who are not able to provide high-quality video recording, as well as those who do not own basic surgical equipment at home, are considered as exclusion criteria. Those who failed to submit a video recording on the appointed time or submitted a video recording in which quality interfere marking process are considered dropout criteria.

### Data collection procedure

On the first day of their surgery rotation virtual learning, participants were introduced to the research, the data collection procedure, assessment instruments and were taught the suturing procedure through webinars by the academic staff of The Surgery Department of The Atma Jaya University. Video recording demonstrated suturing steps with verbal instructions which were generated in-house was also provided in the introduction. They are given 5 days to study the instruction provided in the introduction before uploading a pre-test video recording of themselves performing five simple interrupted sutures to a mannequin. The mannequin was self-generated by participants, using materials readily available at home such as cardboard, cushions or felt. This is considered sufficient as we only measure participants’ knowledge and skills to perform simple interrupted sutures, not the result of the sutures. We included instructions for the participant to fixate the mannequin to minimize movement and ensure a more stable suturing process.

Participants were divided by simple random method into two separate groups and received feedback as well as pre-test marks electronically on day 6. The two groups are distinct from each other by the type of feedback received: (1) The first group received direct verbal feedback from their assessor while both the participant and the assessor simultaneously watch the video sent by the participant. (2) The second group received an email consisting of written feedback, which was given within 24 hours after the assessors received the video sent by the participant. All of the feedbacks are given by assessors who are surgeons with more than 5 years of experience in medical education.

After 6 days of self-study (day 12), participants were required to submit a video recording of themselves performing the same suturing skills and once again was marked the day after. Lastly, we shared an online questionnaire to receive feedback from the participants. The online questionnaire, provided in Appendix Table A1, will be created through the Google form platform, and to be answered anonymously by the participants.

### Assessment

Each of the participants is expected to be marked twice (pre-test and post-test) based on suturing skills checklist and GRS by an assessor who was randomly assigned to prevent bias. Assessors have the responsibility to score participant’s suturing skills based on: (1) Suturing skills checklist which was self-generated and accepted by the academic staff of The Department of Surgery at The School of Medicine and Health Science of Atma Jaya Catholic University of Indonesia and (2) Global Rating Scale (GRS) of suturing skills which was adapted from previous research [6]. The assessment tool has been deeply discussed and was accepted by the whole assessors. Furthermore, training was conducted to make sure assessors have the same perception regarding the assessment tools, and high-quality feedback can be delivered. We had also ensured the perception of a step-by-step approach to simple interrupted sutures is similar among assessors by generating a guide for this as seen in Appendix Table A2. The checklist aims to give an objective evaluation of the handling of suturing material, while the GRS aims to assess how the participant proceeds with the procedure in general, including overall fluency, professionalism and knowledge of the procedure. Each assessor will be assigned to a specific group, in which they will be responsible for providing both checklist and GRS scoring simultaneously.

The suturing skills checklist consists of 8 points, which evaluate two different components (handling of equipment [point 1,2,3 and 7] and suturing skills which include the suturing, knotting and cutting procedure [point 4,5,6 and 8]). Appendix Table A3 presents marks that will be collected in a range of 1–5 followed by submission to the data collector while participants received personal feedbacks (either written or verbal) based on their assessors. Assessors are a lecturer and clinical mentors of The Department of Surgery at The School of Medicine and Health Science of Atma Jaya Catholic University of Indonesia.

### Statistical analysis

Both pre and post-test marks will be collected in numerical data and processed in Statistical Package for the Social Sciences (SPSS version 27, IBM Corp, Armonk, NK, Chicago, Illinois). Data will then be tested for their normality using the Kolmogorov-Smirnov test and Paired T-test will be conducted to analyze normally distributed data, otherwise, the Wilcoxon test will be conducted.

## Result

Data were taken from nine consecutive batches of students participating in the surgery clinical rotation with a total of 199 samples which was divided into two different groups. Out of the 199 samples, 17 subjects (8.5%) were dropped out of the study which was caused by poor video recording quality that complicates the marking process. Five out of the 17 dropout subjects were assigned from the verbal interventional group while 12 others were assigned from the written interventional group. Therefore, the total subjects analyzed in this study were 86 and 96 samples in the verbal and written group respectively or 182 samples cumulatively. The demographic distribution of the two groups is described in Appendix Table A4 as shown below.

The mean values of pre-test and post-test score in suturing skills checklist were 3.98 and 4.66 respectively in the group which received the verbal intervention. While the group which received the written intervention had a mean score of 3.62 and 4.36 for pre-test and post-test respectively. When we break down the mean value into checklist and GRS score separately, we found that the verbal group had a mean checklist pre-test and a post-test score of 4.10 (2.5–5.0) and 4.74 (3.4–5.0) respectively. Furthermore, the verbal group had a mean GRS pre-test and a post-test score of 3.85 (1.40–5.0) and 4.56 (2.4–5.0) respectively. The group which received the written intervention had a mean checklist pre-test and a post-test score of 3.76 (1.9–5.0) and 4.49 (2.9–5.0). The mean score for pre-test and post-test in GRS assessment in the same group was 3.42 (2.0–5.0) and 4.18 (2.4–5.0).

The Kolmogorov-Smirnov test showed that most of the data were normally distributed except for the checklist assessment in the group receiving the written intervention. As the result, paired T-test will be conducted for most of the data group, while the Wilcoxon test will be used to analyze the checklist assessment in the group receiving the written intervention.

The group receiving verbal intervention showed a significant difference in the pre-test (M = 4.11, SD = 0.61) and post-test (M = 4.73, SD = 0.37) of suturing skills checklist; t(85) = −10.63, p = 0.0001. Similarly, the GRS assessment in the same group also showed a significant difference (pre-test [M = 3.83, SD = 1.06] and post-test [M = 4.56, SD = 0.64]); t(86) = −8.10, p = 0.0001. Measurement of effect size was calculated and a score of 1.14 and 0.87 was found at checklist and GRS assessment respectively. Based on the effect size interpretation by Cohen, both the results are considered as having a large effect (>0.8).

Through Wilcoxon test, the group receiving written intervention also showed a significant difference in the pre-test and post-test score of checklist assessment (Z = −7.93, p = 0.0001) and found to be large in its effect size (effect size = 0.8). Through paired T test a significant mean difference was found in the GRS assessment in this group (pre-test [M = 3.42, SD = 0.73] and post-test [M = 4.20, SD = 0.83]); t(105) = −9.62, p = 0.0001, also with a large effect size (effect size = 0.93).

Out of 199 feedback questionnaires shared with the participants, only 53 responded (26.6%). Most of the students conducted self-training before assessment (all participants before pre-test and 92.5% before post-test). A large proportion (62.3%) of participants trained more than five times before pre-test, followed by three to four times trials and one to two times of training with the percentage of 24.5% and 13.2% respectively. As many as 28 participants (52.8%) trained less for post-test compared to pre-test, while no change in the frequency of training was observed in 20 participants (37.8%) and only five (9.4%) increased their frequency of training for post-test. The proportion of participants who trained more than five times before the post-test dropped to around half compared to the number of participants who learned five times before the pre-test. In contrast, the proportion of participants who learned one to two times before post-test increased more than twofold compared to pre-test as presented in Appendix.

49 out of 53 (91.9) agreed that the feedback received were effective. Clear explanation by the assessors was the most common reason with a percentage of 77.1% which was followed by the accuracy of mistakes assessed by the assessor in the second place with 20.8%. In general, 96.3% of participants satisfied with the learning method conducted in the study.

## Discussion

Feedback has been one of the essential parts of medical training. Feedback allows students to maximize their learning potential by not only increasing awareness of their weaknesses and strength but also identification on which improvement can be done [7,8]. Moreover, feedback also had a positive impact on student’s self-directed learning and helps improve communication skills [9,10]. This is in line with the feedback we received from the participants in which 77.1% agrees that assessors were able to provide a clear and thorough explanation on what needs to be improved.

Clinical skills training where students can watch themselves through video recording allow them to self-reflect on their strengths and weaknesses. An addition of feedback by a mentor or facilitator will increase the accuracy and effectiveness in this circumstance [6,11–14]. The findings in our study where both verbal and written intervention provide a significant increase in the checklist and GRS score to support the statement. Both interventions provide a large effect size towards the assessments. Our result also provides a scientific basis that feedback on clinical skills training can be effectively done through virtual learning, especially in this covid-19 pandemic.

Although not assessed in our study, we need to emphasize that feedback might have a different outcome on different individuals. Baadte and Schnotz found that giving feedback to students with a positive academic self-concept would lead to a decline in performance and mood but an increase in effort. In contrast, giving feedback to participants with a negative self-concept would work against the decrease in the mood but did not increase performance and motivation [15]. Many other factors such as frequency and types of feedback also have a role in the effectiveness of feedback [16–18]. How one individual acts towards certain feedback also can be modulated by motivational contexts, such as whether feedback reflects goal achievement, whether learners are oriented toward the informative versus the evaluative aspect of feedback, and whether individual learners are motivated to perform well relative to their peers [19].

Some of our participants may have undergone many clinical rotations and some other just started on the clinical rotation. This may explain why there is a wide difference between pre-test scores between the two groups. However, we do not have the data regarding how many clinical rotations have been done by the participants, therefore we cannot analyze this further.

The difficulties encountered by the participants in maintaining universal and standardized video recording quality are one of the limitations of our study. This also leads to the inability of assessors to score one or more marking components in several participants thus contributing to the dropout rate. Giving away clear and standardized instruction on video recording for instance the height, angle and distance of the camera and adequate lighting might be the solution to tackle this issue. The questionnaire has included the frequency of practice done by each participant. Although so, due to incomplete filling of questionnaires, we were not able to do a statistical analysis on how feedbacks are associated with frequency of practice. Furthermore, we did not analyze for a potential confounding factor such as the frequency and duration of the participants practicing the suturing skills before assessment in which might affect the outcome of improved suturing skills as we expect them to practice as many times as possible to achieve the best learning outcome. Self-reflection done by the participants before submitting the video therefore might include repetitions to obtain the best result could not be controlled either. We expect that the results of our studies will give further insights into the effectiveness of virtual clinical skills learning, especially during this pandemic where virtual learning have been conducted to replace the direct learning process. Although so, further studies with controlled intervention could be conducted to analyze the role of feedback in virtual learning.

## Conclusion

The addition of both verbal or written feedback is proofed to be effective in virtual suturing skills learning in medical students undergoing clinical rotation. Furthermore, a positive response was received from the participants for both feedbacks.

## Figures and Tables

**Figure B1.**
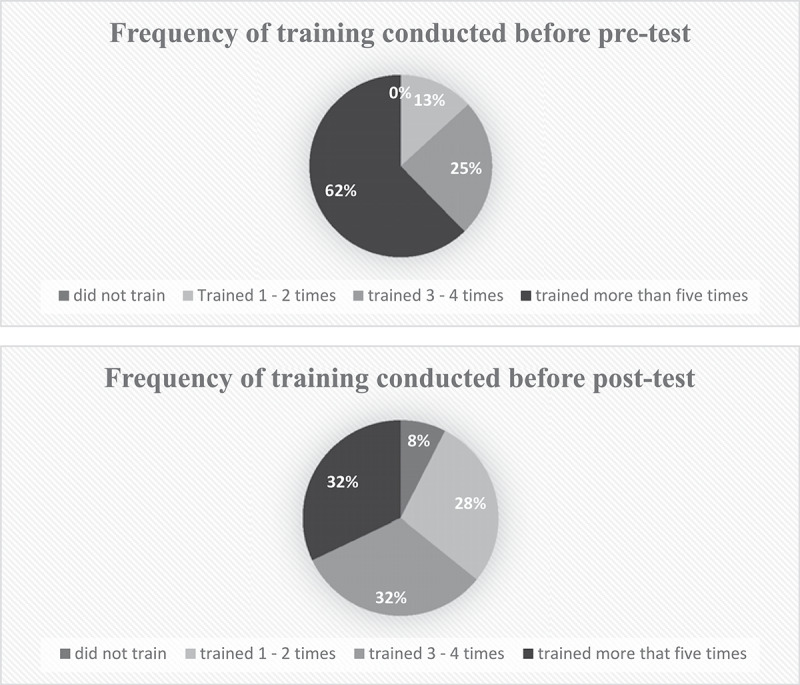
Frequency of training conducted before assessments.

## Abbreviations

SBME- Simulation-Based Medical Education

GRS- Global Rating Scale

## Appendix A. Table

**Table A1.** Intervention feedback questionnaire

Questions	Possible answers				
Did you learn the assessment instrument before submitting the pre-test video?	Yes	No			
Is the assessment instrument help you to do standardize suturing skills?	Yes	No			
Number of training before Pre-test	None	1–2	3–4	≥5	
Number of training before Post-test	None	1–2	3–4	≥5	
Name of assessors					
Is feedback considered useful? (give reason)	Yes	No			
Rate your satisfaction with the learning method (1 being highly unsatisfied, 5 being highly satisfied)	1	2	3	4	5
Please provide feedback for us to improve the learning method					
Is the suturing video shows clear instructions?	Yes	No			
Is the suturing instruction well developed?	Yes	No			
Is the assessment instrument clear and understandable?	Yes	No

**Table A2.** Guide for simple interrupted suturing procedure

Guide for Simple Interrupted Suturing Procedure	
1	Use the needle holder to hold the needle at the needle body, 1/3 proximal from the swaged end
2	The needle is placed at the tip of the needle holder
3	Apply the suture thread with a ratio of 2/3 and 1/3, or use an atraumatic needle if available.
4	Hold the needle holder with your dominant hand, on the distal phalanx of the index and ring finger
5	Hold the forceps with a non-dominant hand, hold it as if you’re holding a pen (use thumb, index and middle finger). Forceps are held at the mid-forceps
6	Place forceps to clamp the edge of the wound, insert needle perpendicular to the mannequin. Note that the hand should be pronated.
7	Advance the needle by pronating the hand, following the curvature of the needle
8	When the tip of the needle is visible on the inner side of the wound, pull out the needle with forceps until the whole needle is outside the wound.
9	Clamp the needle as per instructions 1 to 5
10	Hold the other edge of the wound with the forceps
11	Insert the needle from the inner side of the wound by supinating the hand
12	Once the tip of the needle is seen on the skin, pull it out until the whole needle is outside the skin
13	Pull the suture thread until you reach the desired length. You now have a long end on one side and a short end on the other
14	Place the forceps and needle on sterile area
15	Hold the long end of the suture thread with a non-dominant hand, while the dominant hand is holding the needle holder
16	Place the needle holder in between the two suture threads
17	Make a surgical knot on the needle holder by encircling the long end of the suture thread on the needle holder twice
18	With the needle holder, clamp the short end of the suture thread
19	Pull the short end of the suture thread opposite to the original place (the short end of the suture thread is pulled to the side of the long end of the suture thread). Now you have the first knot.
20	For the second knot, first place the needle holder is placed in between the suture thread
21	Encircle the long end of the suture thread once on the needle holder
22	With the needle holder, clamp the short end of the suture thread
23	Pull the short end of the suture thread opposite to the original place (the short end of the suture thread is pulled to the side of the long end of the suture thread)
24	Place the knot on either edge of the wound
25	The suture should be a non-tension suture. Edges of the wound are expected to be right next to each other and should not be inverted or overlapped
26	Pull the two ends of the needle thread with a non-dominant hand, while the dominant hand hold the thread scissors
27	Hold the thread scissors with the distal phalanx of the thumb and ring fingers
28	Cut the excessive suture thread with the tip of the thread scissors.
29	Suture threads are cut approximately 1 cm above the knot, cut with a 45° angle.

**Table A3.** Assessment tools

No.	Variable	Rating					
	Suturing Checklist	1	2	3	4		5
1.	Handling of the needle holder	Mostly incorrect	75% incorrect	50% incorrect	75% correct		Mostly correct
2.	Handling of tissue forceps						
3.	Loading of the needle to the needle holder						
4.	Piercing the skin surface						
5.	Pulling needle from the tissue						
6.	Knotting						
7.	Handling of surgical scissor						
8.	Cutting						
	Global Rating Scale	1		3		5	
1.	Respect for tissue	Often used unnecessary force on tissue or caused damage by inappropriate use of instruments		Careful handling of tissue but occasionally caused inadvertent damage		Consistently handled tissues appropriately, with minimal damage	
2.	Time and motion	Many unnecessary moves		Efficient time and motion, but some unnecessary moves		The economy of movement and maximum efficiency	
3.	Instrument handling	Repeatedly makes tentative or awkward moves with instruments		Competent use of instruments, although occasionally appeared stiff or awkward		Fluid moves with instruments and no awkwardness	
4.	Knowledge of instruments	Frequently asked for the wrong instrument or used an inappropriate instrument		Knew the names of most instruments and used appropriate instruments of the task		Obviously familiar with the instruments required and their names	
5.	The flow of operation and forward planning	Frequently stopped operating or needed to discuss next move		Demonstrated ability for forward planning with a steady progression of the operative procedure		Obviously planned course of operation with effortless flow from one move to the next	
6.	Knowledge of Specific Procedure	Deficient knowledge. Needed specific instructional most operative steps		Knew all important aspects of the operation		Demonstrated familiarity with all aspects of the operation

**Table A4.** Demographical data of the participants

	Gender		TOTAL
Interventional Groups	Male	Female	
Verbal	36	50	86
Written	30	66	96
TOTAL	66 (36.3%)	116 (63.7%)	182
